# First Insights into the Genome Sequence of *Pseudomonas oleovorans* DSM 1045

**DOI:** 10.1128/genomeA.00774-17

**Published:** 2017-08-10

**Authors:** Anja Poehlein, Rolf Daniel, Andrea Thürmer, Alexander Bollinger, Stephan Thies, Nadine Katzke, Karl-Erich Jaeger

**Affiliations:** a Genomic and Applied Microbiology & Göttingen Genomics Laboratory, Georg-August University Göttingen, Göttingen, Germany; b Institute of Molecular Enzyme Technology, Heinrich-Heine-University Düsseldorf, Jülich, Germany; c Institute of Bio- and Geosciences IBG-1: Biotechnology, Forschungszentrum Jülich GmbH, Jülich, Germany

## Abstract

The Gram-negative proteobacterium *Pseudomonas oleovorans* DSM 1045 is considered a promising source for enzymes of biotechnological interest, e.g., hydrolases and transaminases. Here, we present a draft sequence of its 4.86-Mb genome, enabling the identification of novel biocatalysts.

## GENOME ANNOUNCEMENT

Hydrocarbon-degrading bacteria, including members of the genus *Pseudomonas*, represent a promising source for novel biocatalysts of biotechnological relevance (1, 2). Belonging to this group, the *Pseudomonas oleovorans* type strain DSM 1045 was isolated as a contaminant of industrial cutting fluids and shown to utilize cyclic aliphatic hydrocarbons, like naphtenic acids (2). Its biotechnological potential was indicated by the observation that cell extracts could catalyze ω-transamination reactions (3).

Chromosomal DNA of *Pseudomonas oleovorans* DSM 1045 was isolated from 2 ml of overnight-grown culture (growth medium LB, 30°C; Carl-Roth-Karlsruhe) using the DNeasy blood and tissue kit (Qiagen, Hilden, Germany), according to the manufacturer’s instructions. The extracted DNA was used to generate Illumina shotgun paired-end sequencing libraries, which were sequenced with a MiSeq instrument and the MiSeq reagent kit version 3 (600 cycles), as recommended by the manufacturer (Illumina, San Diego, CA, USA). Quality filtering using Trimmomatic version 0.32 (4) resulted in 2,602,096 paired-end reads. The assembly was performed with the SPAdes genome assembler software version 3.8.0 (5) and resulted in 108 contigs (>500 bp), with an average coverage of 112-fold. The assembly was validated and the read coverage determined with QualiMap version 2.1 (6). The draft genome of *P. oleovorans* DSM 1045 consisted of a single chromosome (4.86 Mb) with an overall G+C content of 62.07%. Automatic gene prediction and identification of rRNA and tRNA genes were performed using the software tool Prokka (7). The draft genome contained 7 rRNA genes, 62 tRNA genes, 3,398 protein-coding genes with predicted functions, and 1,243 genes coding for hypothetical proteins.

A homology search for biocatalysts of potential biotechnological relevance with all *in silico*-translated coding sequences (CDSs) using BLASTP (8) led to the detection of 15 putative enzymes predicted to be lipases, esterases, or phospholipases. Furthermore, three putative ω-transaminases were identified, and one imine reductase was identified according to sequence motifs described in Fademrecht et al. (9). Genes encoding a Sec and Tat secretion pathway, as well as genes encoding a type II secretion machinery, were identified, indicating the potential to produce extracellular enzymes. Furthermore, biosynthetic capabilities are predicted for antimicrobial bacteriocins and polyhydroxyalkanoate biopolymers, as identified with antiSMASH 4.0.0rc1 (10). An aliphatic alkane degradation pathway could not be detected, coinciding with the observation that this strain does not grow on long-chain alkanes (11). Genes encoding homologs to aliphatic alcohol dehydrogenase AlkJ (of *Pseudomonas putida* GPo1) and rhamnosyltransferase RhlA (of *Pseudomonas aeruginosa*) further suggest capabilities for the synthesis of aliphatic alcohols and of 3-(hydroxyalkanoyloxy)alkanoic acid type biosurfactants.

### Accession number(s).

This whole-genome shotgun project has been deposited at DDBJ/ENA/GenBank under the accession no. NIUB00000000. The version described in this paper is version NIUB01000000.
